# Impact of Neoadjuvant Chemotherapy and Preoperative Irradiation on Early Complications in Direct-to-Implant Breast Reconstruction

**DOI:** 10.1055/a-2358-8864

**Published:** 2024-08-06

**Authors:** Ji Won Hwang, Su Min Kim, Jin-Woo Park, Kyong-Je Woo

**Affiliations:** 1 Department of Plastic and Reconstructive Surgery, Ewha Womans University Mokdong Hospital, Seoul, South Korea; 2 Department of Plastic Surgery, Samsung Medical Center, Sungkyunkwan University School of Medicine, Seoul, South Korea

**Keywords:** direct-to-implant reconstruction, complications, neoadjuvant chemotherapy, prior radiation history

## Abstract

**Background** Impact of previous radiation therapy and neoadjuvant chemotherapy (NACT) on early complications in direct-to-implant (DTI) breast reconstruction has not been elucidated. This study investigated whether DTI reconstruction is viable in patients with NACT or a history of preoperative chest wall irradiation.

**Methods** Medical records of breast cancer patients who underwent nipple-sparing or skin-sparing mastectomy with DTI breast reconstruction from March 2018 to February 2021, with at least 1 year of follow-up in a single tertiary center, were reviewed. Demographic data, intraoperative details, and postoperative complications, including full-thickness necrosis, infection, and removal, were reviewed. Risk factors suggested by previous literature, including NACT and preoperative chest wall irradiation histories, were reviewed by multivariate analysis.

**Results** A total of 206 breast cancer patients were included, of which, 9 were bilateral, 8 patients (3.9%) had a history of prior chest wall irradiation, and 17 (8.6%) received NACT. From 215 cases, 11 cases (5.1%) required surgical intervention for full-thickness necrosis, while intravenous antibiotics or hospitalization was needed in 11 cases (5.1%), with 14 cases of failure (6.5%) reported. Using multivariable analysis, preoperative irradiation was found to significantly increase the risk of full-thickness skin necrosis (OR = 12.14, *p* = 0.034), and reconstruction failure (OR = 13.14, *p* = 0.005). NACT was not a significant risk factor in any of the above complications.

**Conclusion** DTI breast reconstruction is a viable option for patients who have received NACT, although reconstructive options should be carefully explored for patients with a history of breast irradiation.

## Introduction

Implant-based breast reconstruction is the most common reconstructive surgery following a mastectomy. Improvements in surgical techniques and technological advances have made direct-to-implant (DTI) insertion a much more viable option. 1 However, DTI is not without risk of complications, which range from skin necrosis, seroma, infection, and loss of implants. Predictable risk factors to identify the increased complications have been proposed in previous literature, such as smoking, obesity, and bigger breast/implant sizes. 2 Although fears of perioperative or postoperative complications for breast reconstruction after neoadjuvant chemotherapy (NACT) and prior irradiation have been proposed, studies have produced mixed results.

NACT is frequently administered to downstage the tumor and limit the extent of axillary lymph node removal. 3 However, NACT can compromise the immunogenicity and the tissue healing capacity, causing a predisposition to infection or dehiscence. 4 Yet, studies have called into question the allegedly harmful relationship between NACT and immediate breast reconstruction. 5 Some women with a history of breast conservation surgery and radiation later undergo mastectomy, either for recurrence or genetic predisposition. The estimated rates of recurrence after breast conserving surgery (BCS) range between 8 and 14% over a 20-year period. 6 Radiation exposure produces fibrosis and vascular thickening of the skin and subcutaneous tissues, which makes the irradiated breast susceptible to adverse clinical outcomes after reconstruction. 7 Still, DTI breast reconstruction is an option for many women, even after salvage mastectomy. 8 However, the selection of autologous versus implant breast reconstruction in these patients remains controversial.

As DTI is becoming one of the most selected reconstructive options for many prospective patients; therefore, a better understanding of the evidence-based comparative risks related to reconstruction options is needed to further inform the shared decision-making. Complication rates remain higher in radiated breasts, even with autologous tissue, and some patients prefer implant-based reconstruction (IBR), while some cannot be considered candidates for autologous reconstruction. 9 Previous studies that have examined the relationship between NACT and the outcomes of breast reconstruction have focused primarily on autologous reconstruction or two-stage reconstruction with prostheses. DTI can be different in terms of complications because it does not undergo skin expansion. Furthermore, evidence-based reports related to their independent effects on morbidity after mastectomy with DTI breast reconstruction are lacking or limited by small sample sizes. Our objective was to determine whether DTI is a viable reconstructive option in patients with NACT or a prior history of irradiation, as well as to identify factors for complications of suboptimal implant reconstruction results.

## Methods

### Data Collection

Medical records of breast cancer patients who underwent nipple-sparing or skin-sparing mastectomy (SSM) with immediate breast reconstruction with DTI, from March 2018 to February 2021, and with at least 1 year of follow-up in a single tertiary center were reviewed. This retrospective cohort study was approved by the Institutional Review Board of the author's institution (No. 2023-02-023). Demographic data, intraoperative details, and major postoperative complications, including full-thickness necrosis, infection, seroma, and reconstruction failure were collected. Risk factors suggested in previous literature, 10 including NACT and preoperative chest wall irradiation history, were reviewed using multivariate analysis. Major complications were defined as follows: full-thickness necrosis requiring surgical intervention, infection requiring intravenous (IV) antibiotics or hospitalization, seroma requiring aspiration or documented radiologically, and implant extrusion. The need for surgical intervention or hospitalization was determined by the senior author.

### Surgical Technique

Mastectomy was performed by eight surgical oncology specialists, while all reconstructions were performed in single-stage, using DTI insertion. The size of the implant was determined by the patient's goals, breast width, and mastectomy weight. The implant (BellaGel microtextured round implants [Hans Biomed Corp, Korea], microtextured anatomical implant [Mentor, Santa Barbara, CA], or smooth round implant [Mentor, Santa Barbara, CA]) was placed either prepectorally or subpectorally, which was determined by the condition of the mastectomy skin flap. Acellular dermal matrix (MegaDerm; L&C Bio, South Korea; CGderm; CGBIO, Inc., Seongnam, South Korea; or CG CRYODERM; CGBIO, Inc., Seongnam, South Korea) was used in all cases, either by fully wrapping the implant or suturing it to the inferolateral border of the pectoralis major muscle to cover the implant's lower pole. Either one or two closed-suction drains were placed, with reference to the size of the breast and the plane of implant placement.

### Statistical Analysis

The main aim of the analysis was to evaluate the association between any complications and the following variables: body mass index, smoking status, mastectomy weight, implant size, type of axillary surgery, the plane of implant insertion, and comorbidities, including diabetes and hypertension, aside from NACT and prior radiation history. Univariate and multivariable logistic regression analyses were performed by adjusting for possible risk factors for each major complication. The Statistical Package for the Social Sciences (SPSS version 21; IBM Co., Armonk, NY) was used for data analysis. The significance level was set at *p* < 0.05 (two-sided). Continuous data are expressed as the mean ± standard deviation, and categorical data are expressed as sample numbers and percentages.

## Results

The study population included 206 breast cancer patients, which comprised 9 bilateral, 17 (8.6%) who had received NACT, and 8 (3.9%) with a prior history of chest wall irradiation. The mean BMI of patients was 22.6 ± 2.9 kg/m ^2^ , and most had medium-sized breasts with a mean mastectomy weight of 252 g (176–352 g) and a mean implant size of 274.83 ± 98.30 cc. A total of six patients (2.9%) were active or former smokers. A total of 127 cases (59.1%) of the implants were placed prepectorally, and 80% of the patients had only undergone sentinel lymph node biopsy. The demographic data of patients are summarized in Table 1 .

**Table 1:** Demographics

	*N* = 206 (215 breasts)
Age, years (mean ± SD)	50.67 ± 8.51
Body mass index, kg/m ^2^ (mean ± SD)	22.64 ± 2.93
Implant size, mL (mean ± SD)	274.83 ± 98.30
Mastectomy weight, g (mean ± SD)	252 (176–352)
Smoking, number (%)	
Never	200 (97.1%)
Active or former	6 (2.9%)
Diabetes, number (%)	10 (4.9%)
Hypertension, number (%)	27 (13.1%)
Preoperative radiation therapy, number (%)	8 (3.9%)
Preoperative chemotherapy, number (%)	17 (8.3%)
Adjuvant radiation therapy, number (%)	36 (17.8%)
Adjuvant chemotherapy, number (%)	78 (38.6%)
Implant plane of insertion	
Prepectoral, number (%)	127 (59.1%)
Subpectoral, number (%)	88 (40.9%)
Axillary surgery	
Sentinel lymph node biopsy	172 (80.0%)
Axillary lymph node dissection	43 (20.0%)

Abbreviation: SD, standard deviation.

From 215 cases, 11 cases (5.1%) required surgical intervention for full-thickness necrosis, while IV antibiotics or hospitalization were needed in 11 cases (5.1%), 14 cases seroma (6.5%) that required aspiration or were documented by radiology occurred, and 14 cases were reported for failure (6.5%; Table 2 ).

**Table 2:** Rate of complications

	*N* = 215
Skin flap complication requiring surgical intervention, *N* (%)	11 (5.1%)
Infection, *N* (%)	11 (5.1%)
Seroma, *N* (%)	14 (6.5%)
Device removal/exchange, *N* (%)	14 (6.5%)

Using multivariable analysis, preoperative irradiation was found to significantly increase the risk of full-thickness skin necrosis (OR = 12.14, *p* = 0.034) and implant failure (OR = 13.14, *p* = 0.005). NACT was found to not be a significant risk factor in any of the above complications ( Table 3 ).

Full-thickness necrosis, which required surgical intervention was significantly associated with preoperative radiation therapy in the univariate analysis ( *p* = 0.024) and with implant plane of insertion ( *p* = 0.04). Both were significant when controlling for other risk factors, with an odds ratio of 12.141 for preoperative radiation and 6.457 for the subpectoral plane of insertion ( Table 3.1 ).

**Table 3:** Univariable and multivariable analyses for skin necrosis, infection, and reconstruction failure

**3.1:** Full thickness necrosis

	Univariate analysis				Multivariate analysis			
	Odds ratio	Lower 95% CI	Upper 95% CI	*p* -Value	Odds ratio	Lower 95% CI	Upper 95% CI	*p* -Value
Body mass index	1.016	0.828	1.247	0.880	1.050	0.756	1.457	0.772
Smoking (current or ex-smoker vs. never smoker)	NA	NA	NA	NA	NA	NA	NA	NA
Diabetes (yes vs. no)	NA	NA	NA	NA	NA	NA	NA	NA
Hypertension (yes vs. no)	NA	NA	NA	NA	NA	NA	NA	NA
Preoperative radiation therapy (yes vs. no)	7.333	1.295	41.536	0.024 ^a^	12.141	1.200	122.836	0.034 ^a^
Preoperative chemotherapy (yes vs. no)	1.175	0.141	9.770	0.881	0.566	0.032	9.981	0.697
Axillary surgery type (ALND vs. SLNB)	1.537	0.390	6.058	0.539	0.741	0.098	5.624	0.772
Mastectomy weight	0.561	0.165	1.906	0.354	1.005	0.998	1.012	0.183
Implant size	1.002	0.999	1.005	0.174	0.993	0.982	1.005	0.246
Implant insertion plane (subpectoral vs. prepectoral)	4.133	1.065	16.045	0.040 ^a^	6.457	1.367	30.495	0.019 ^a^
Adjuvant chemotherapy (yes vs. no)	1.958	0.578	6.636	0.281	1.265	0.262	6.102	0.770
Adjuvant radiation therapy (yes vs. no)	1.831	0.462	7.258	0.389	1.575	0.245	10.136	0.632

Abbreviations: ALND, axillary lymph node dissection; NA, not applicable; SLND, sentinel lymph node biopsy.

Infections that required IV antibiotics or hospitalization were significantly associated with preoperative radiation ( *p* = 0.024) and mastectomy weight ( *p* = 0.017) following univariate analysis. Preoperative radiation was associated with increased odds of infection with borderline significance in multivariable analysis ( *p* = 0.070). There were no other significant factors for increased risk of infection after multivariate analysis ( Table 3.2 ).

**3.2:** Infection

	Univariate analysis				Multivariate analysis			
	Odds ratio	Lower 95% CI	Upper 95% CI	*p* -Value	Odds ratio	Lower 95% CI	Upper 95% CI	*p* -Value
Body mass index	1.068	0.881	1.294	0.504	0.940	0.693	1.275	0.689
Smoking (current or ex-smoker vs. never smoker)	NA	NA	NA	NA	NA	NA	NA	NA
Diabetes (yes vs. no)	NA	NA	NA	NA	NA	NA	NA	NA
Hypertension (yes vs. no)	NA	NA	NA	NA	NA	NA	NA	NA
Preoperative radiation therapy (yes vs. no)	7.333	1.295	41.536	0.024	6.384	0.862	47.260	0.070
Preoperative chemotherapy (yes vs. no)	1.175	0.141	9.770	0.881	0.818	0.072	9.234	0.871
Axillary surgery type (ALND vs. SLNB)	1.537	0.390	6.058	0.539	0.788	0.123	5.048	0.801
Mastectomy weight	1.003	1.001	1.006	0.017	1.006	1.000	1.013	0.061
Implant size	1.004	0.998	1.010	0.245	0.996	0.985	1.008	0.533
Implant insertion plane (subpectoral vs. prepectoral)	0.525	0.135	2.037	0.352	0.570	0.129	2.521	0.458
Adjuvant chemotherapy (yes vs. no)	2.917	0.826	10.297	0.096	1.533	0.311	7.552	0.599
Adjuvant radiation therapy (yes vs. no)	2.892	0.801	10.442	0.105	2.620	0.471	14.590	0.271

Abbreviations: ALND, axillary lymph node dissection; NA, not applicable; SLND, sentinel lymph node biopsy.

No adjusted variables were associated with seroma in either the univariate or multivariate analysis ( Table 3.3 ).

**3.3:** Seroma

	Univariate analysis				Multivariate analysis			
	Odds ratio	Lower 95% CI	Upper 95% CI	*p* -Value	Odds ratio	Lower 95% CI	Upper 95% CI	*p* -Value
Body mass index	1.161	0.989	1.362	0.069	1.046	0.828	1.320	0.707
Smoking (current or ex-smoker vs. never smoker)	NA	NA	NA	NA	NA	NA	NA	NA
Diabetes (yes vs. no)	4.021	0.768	21.053	0.099	2.515	0.342	18.501	0.365
Hypertension (yes vs. no)	2.95	0.858	10.148	0.086	2.710	0.604	12.147	0.193
Preoperative radiation therapy (yes vs. no)	2.132	0.244	18.657	0.494	2.964	0.286	30.733	0.363
Preoperative chemotherapy (yes vs. no)	0.889	0.109	7.242	0.913	1.321	0.103	17.020	0.831
Axillary surgery type (ALND vs. SLNB)	1.662	0.495	5.578	0.411	2.096	0.446	9.845	0.349
Mastectomy weight	1.002	0.999	1.004	0.226	1.001	0.997	1.006	0.570
Implant size	1.004	0.998	1.009	0.168	1.001	0.993	1.010	0.771
Implant insertion plane (subpectoral vs. prepectoral)	0.557	0.169	1.837	0.337	0.499	0.137	1.814	0.291
Adjuvant chemotherapy (yes vs. no)	1.194	0.399	3.575	0.751	1.045	0.288	3.789	0.946
Adjuvant radiation therapy (yes vs. no)	0.771	0.165	3.602	0.741	0.624	0.083	4.694	0.647

Abbreviations: ALND, axillary lymph node dissection; SLND, sentinel lymph node biopsy.

A significant association was found between prior irradiation and reconstruction failure in the univariate analysis ( *p* = 0.003), while an association with axillary lymph node dissection was less marked ( *p* = 0.035). Using multivariate analysis, only an association with preoperative radiation therapy was shown to be significant, which indicated a 13.1 times higher risk of failure ( Table 3.4 ).

**3.4:** Reconstruction failure

	Univariate analysis				Multivariate analysis			
	Odds ratio	Lower 95% CI	Upper 95% CI	*p* -Value	Odds ratio	Lower 95% CI	Upper 95% CI	*p* -Value
Body mass index	1.089	0.919	1.289	0.325	0.997	0.783	1.269	0.982
Smoking (current or ex-smoker vs. never smoker)	NA	NA	NA	NA	NA	NA	NA	NA
Diabetes (yes vs. no)	1.641	0.193	13.961	0.650	2.605	0.213	31.896	0.454
Hypertension (yes vs. no)	0.496	0.062	3.944	0.507	0.463	0.046	4.658	0.513
Preoperative radiation therapy (yes vs. no)	10.691	2.258	50.615	0.003	13.140	2.207	78.246	0.005 ^a^
Preoperative chemotherapy (yes vs. no)	3.643	0.910	14.586	0.068	2.106	0.341	12.992	0.423
Axillary surgery type (ALND vs. SLNB)	3.324	1.088	10.158	0.035	3.888	0.854	17.700	0.079
Mastectomy weight	1.002	0.999	1.005	0.195	1.001	0.996	1.007	0.615
Implant size	1.003	0.998	1.009	0.205	1.002	0.993	1.012	0.632
Implant insertion plane (subpectoral vs. prepectoral)	0.790	0.255	2.442	0.682	0.738	0.205	2.660	0.642
Adjuvant chemotherapy (yes vs. no)	1.627	0.549	4.821	0.380	0.858	0.229	3.211	0.820
Adjuvant radiation therapy (yes vs. no)	1.988	0.588	6.722	0.269	0.887	0.172	4.571	0.886

Abbreviations: ALND, axillary lymph node dissection; SLND, sentinel lymph node biopsy.

When conducting multiple logistic regression analysis using “stepwise selection” as the variable section method, prior irradiation (odds = 11.276, *p* = 0.013) and subpectoral placement of the implant (odds = 5.188, *p* = 0.026) emerged as significant risk factors for full-thickness necrosis of the skin flap. Additionally, prior irradiation (odds = 13.562, *p* = 0.002) and axillary lymph node dissection (odds = 3.940, *p* = 0.024) were identified as significant risk factors for reconstruction failure ( Table 4 ).

**Table 4:** Multivariable analysis of risk factors for skin flap necrosis and reconstruction failure a

	Full thickness necrosis of skin flap		Reconstruction failure	
	Odds ratio (95% CI)	*p* -Value	Odds ratio (95% CI)	*p* -Value
Preoperative radiation therapy	11.276 (1.659–76.624)	0.013	13.562 (2.625–70.074)	0.002
Implant insertion plane (subpectoral vs. prepectoral)	5.188 (1.214–22.159)	0.026	–	–
Axillary surgery type (ALND vs. SLNB)	–	–	3.940 (1.198–12.962)	0.024

Abbreviations: ALND, axillary lymph node dissection; SLND, sentinel lymph node biopsy.

a “Stepwise selection” was used for variable selection for multiple logistic regression analysis.

## Discussion

The current study demonstrated that a history of radiation therapy significantly increases the risk of mastectomy flap necrosis, and reconstruction failure in DTI, whereas NACT was not an independent risk factor for any of the complications explored in this study. This result suggests that the immediate reconstruction of the breast with a prosthesis is a viable option for patients who have previously received NACT; however, reconstructive options should be carefully explored for patients with a history of breast irradiation.

NACT was originally offered to patients with locally advanced breast cancer, although it is now utilized more often, resulting in approximately 16 to 17% of patients converting from mastectomy to BCS. 11 Performing IBR after NACT is generally considered safe, 12 13 14 although there have been contradictory reports. Varghese et al reported a significant increase in implant/expander loss after NACT and a trend toward increased postoperative complications, 15 while Frey et al reported an increased risk of implant loss in the NACT group. 16 However, the examinations conducted in the previous literature contain limitations in examining the effect of NACT in the DTI group only with SSM, 17 two-staged reconstruction group, 5 and a mixed cohort of implant-based and autologous reconstruction. 18 This study comprised patients solely receiving DTI after nipple sparing mastectomy or SSM, which proved this reconstructive method as being safe in patients with NACT.

The deleterious effects of postmastectomy radiotherapy on reconstruction have been previously well-documented. 19 However, the safety of performing IBR after prior radiotherapy remains controversial; McCarthy et al reported two-staged IBR to be a viable option in patients with a history of radiotherapy, 20 whereas Spear et al argued successful two-staged IBR to be the exception. 21 Hirsch et al found a 60% chance of success when using two-staged IBR in patients who had received prior radiation, 22 and insisted on a frank discussion with the patient regarding the reconstructive outcomes. This study is the first to explore the safety of DTI in patients with a history of irradiation. Our study examined the effect of prior radiation on DTI and found a similarly increased risk of postoperative complications and reconstructive failure. Considering the high success rate of autologous reconstruction, even with a history of radiation, 23 careful consultations with the patient regarding respect to risks and alternatives to reconstructive surgery are needed in patients with a history of breast irradiation.

This study is original in its analysis of DTI as the sole reconstructive option in patients with a history of NACT or radiation. Many of the earlier studies analyzing the effect of NACT or prior radiation have often included a composite group of reconstructive techniques, including autologous reconstruction or two-staged IBRs. 24 Much of the previous literature has included cases of subpectoral implant insertion; however, more than half of the cases in this study used prepectoral plane insertion. The complication rates in this study are within the ranges reported in previous literature, 25 although this study was limited by its homogeneous population, which possessed relatively small- to medium-sized breasts.

Indocyanine green angiography was routinely used in our institution from the middle of 2018 to assess mastectomy skin flap perfusion, and poor perfusion was used as one of the indications to insert implants subpectorally. This could explain why the plane of implant insertion was a significant factor for an increased risk of mastectomy flap necrosis.

Given the small sample size of patients with a history of prior irradiation or NACT, we decided not to pursue a comparative analysis but instead implemented an analysis of the incidence of complications in order to establish their association with different variables. All eight patients with a history of prior chest wall irradiation had radiation due to breast cancer treated with breast-conserving therapy and were treated with completion mastectomy and reconstruction with prosthesis from recurrence. Considering the low recurrence rate after BCS and radiation therapy, or the likelihood of receiving BCS after NACT, the absolute number of patients with a history of radiation or NACT was low, despite observing more than 200 patients over a 3-year period. However, a history of radiation proved to significantly affect the risk of complications after DTI in both the univariate and multivariate analyses controlling for adjuvant treatment modalities as well.

### Conclusion

When discussing potential DTI reconstruction with patients who have a history of prior breast irradiation, the patient should be counseled on the high likelihood of postoperative complications and reconstructive failures. Although DTI can be safely recommended in patients with NACT, patients with a history of radiation who truly understand the risks of DTI and yet opt to not undergo autologous reconstruction should be offered this choice.
